# Supplementary figures and images for: Limited efficacy of repeated praziquantel treatment in Schistosoma mansoni infections as revealed by highly accurate diagnostics, PCR and UCP-LF CAA (RePST trial)
Source: PLoS Negl Trop Dis. 2022 Dec 22;16(12):e0011008. doi: 10.1371/journal.pntd.0011008 (PMC9822103; doi:10.1371/journal.pntd.0011008)

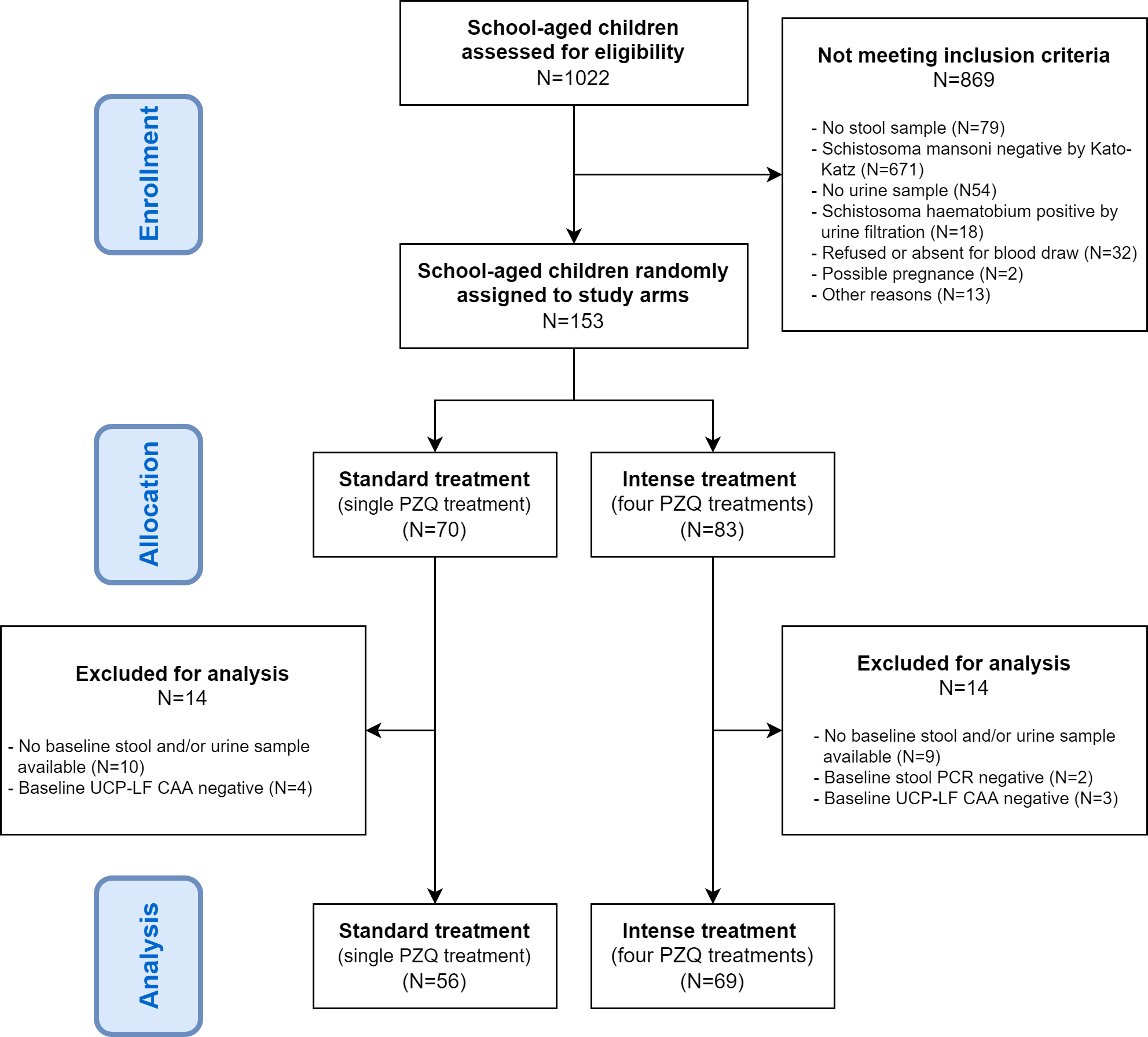

Supplement: S1 Fig — (PNG) [file pntd.0011008.s001.png]

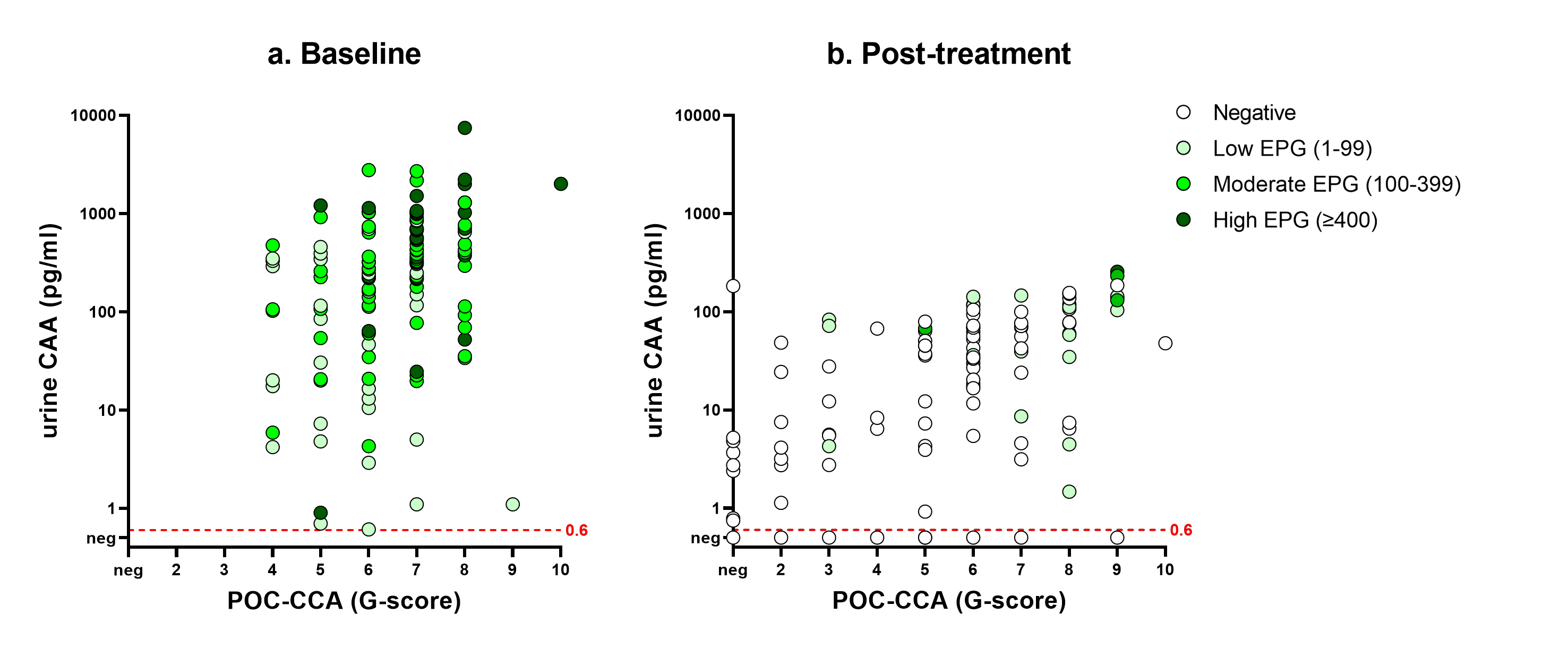

Supplement: S2 Fig — Correlation between worm-based detection methods including intensity categories based on Kato-Katz (eggs per gram of feces, EPG) at (a) baseline and (b) 4 weeks after (the last) treatment. Data based on point-of-care circulating cathodic antigen (POC-CCA) and up-converting particle circulating anodic antigen (UCP-LF CAA) in combination with Kato-Katz (KK), with colors indicating the EPG intensity (n = 125). (TIF) [file pntd.0011008.s002.tif]

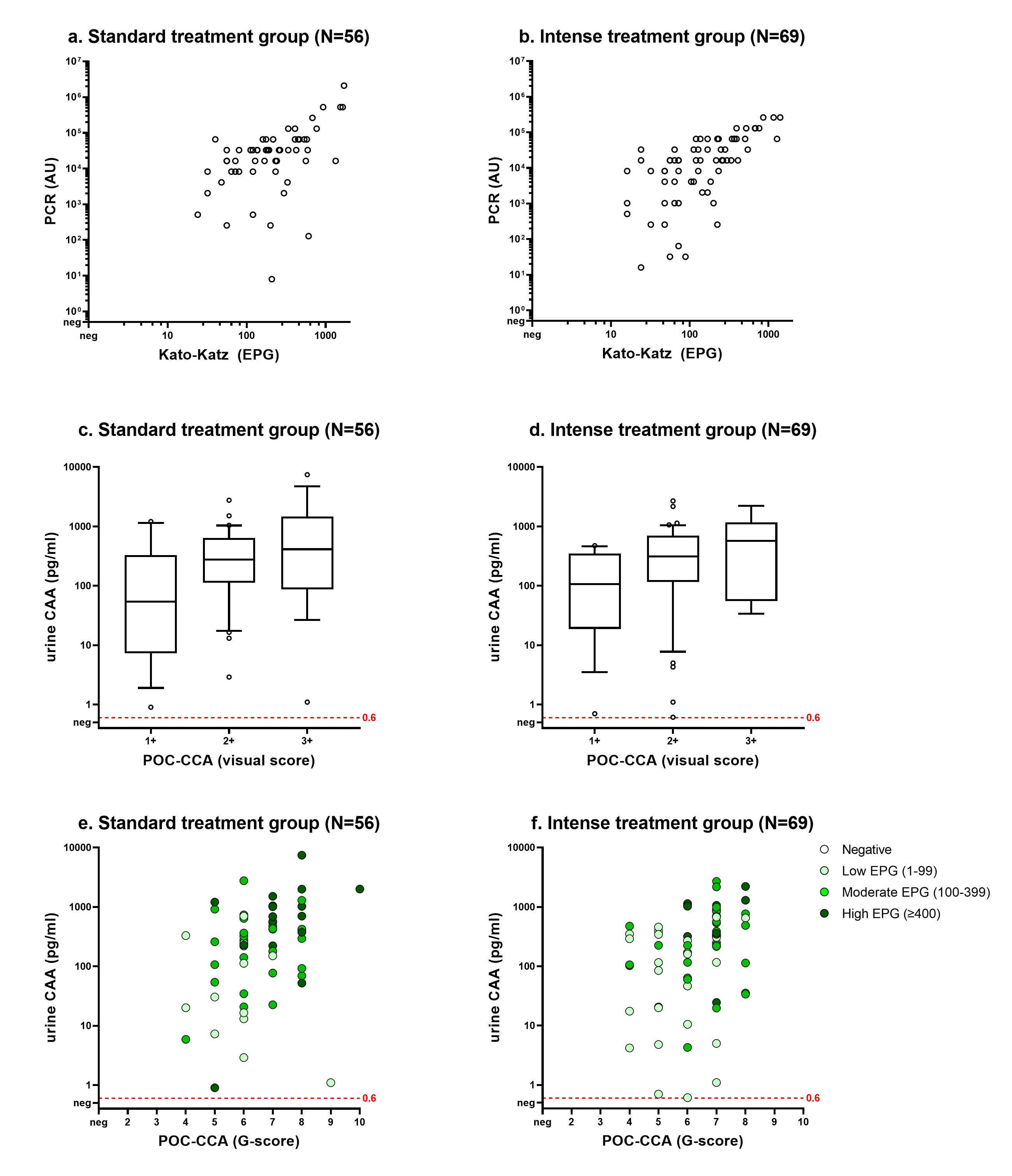

Supplement: S3 Fig — Data based on stool polymerase chain reaction (PCR) versus Kato-Katz (KK) (a-b) and point-of-care circulating cathodic antigen (POC-CCA) versus up-converting particle circulating anodic antigen (UCP-LF CAA) (c-f) at baseline (n = 125). (TIF) [file pntd.0011008.s003.tif]

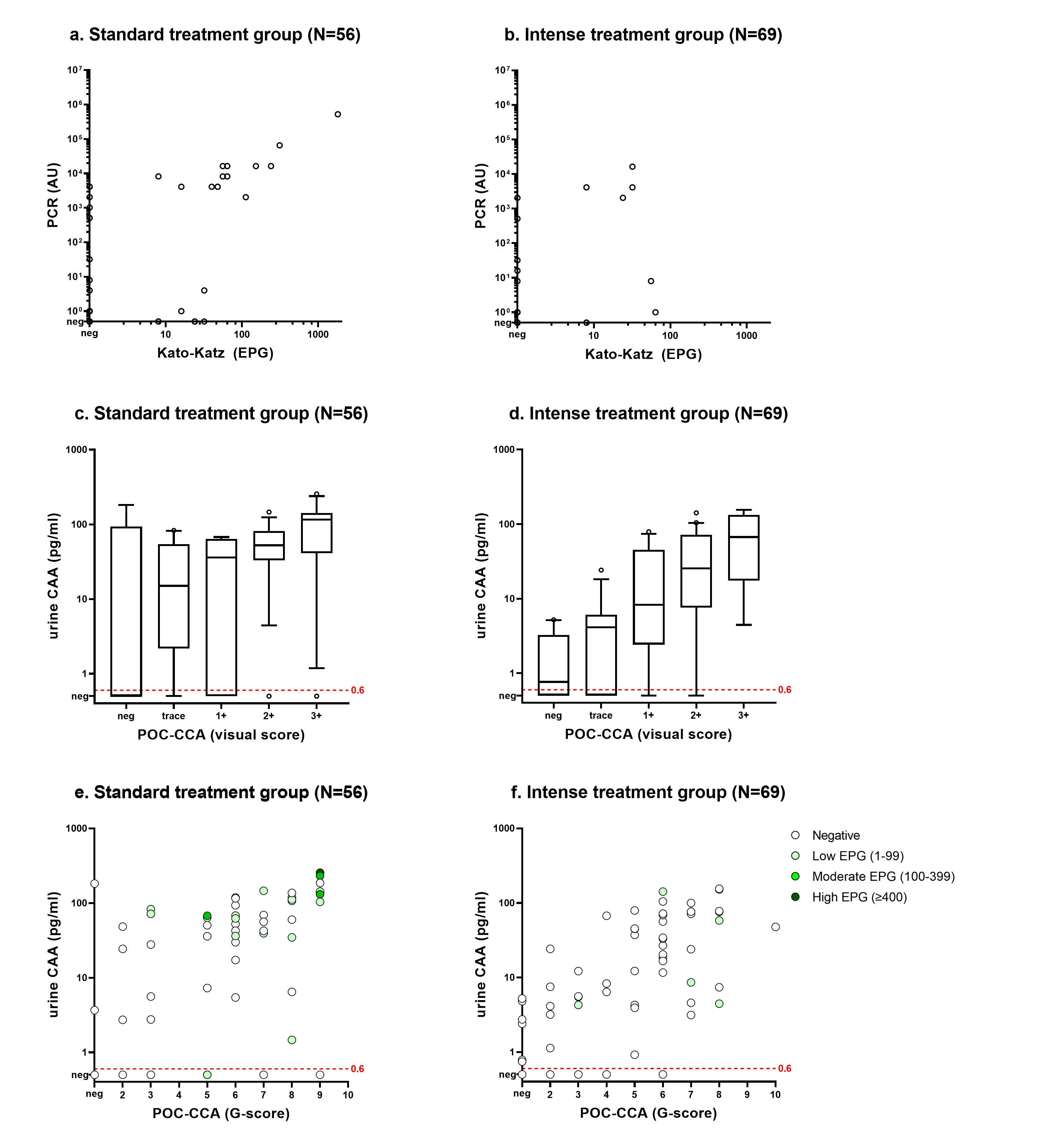

Supplement: S4 Fig — Data based on stool polymerase chain reaction (PCR) versus Kato-Katz (KK) (a-b) and point-of-care circulating cathodic antigen (POC-CCA) versus up-converting particle circulating anodic antigen (UCP-LF CAA) (c-f) 4 weeks post-treatment (n = 125). (TIF) [file pntd.0011008.s004.tif]

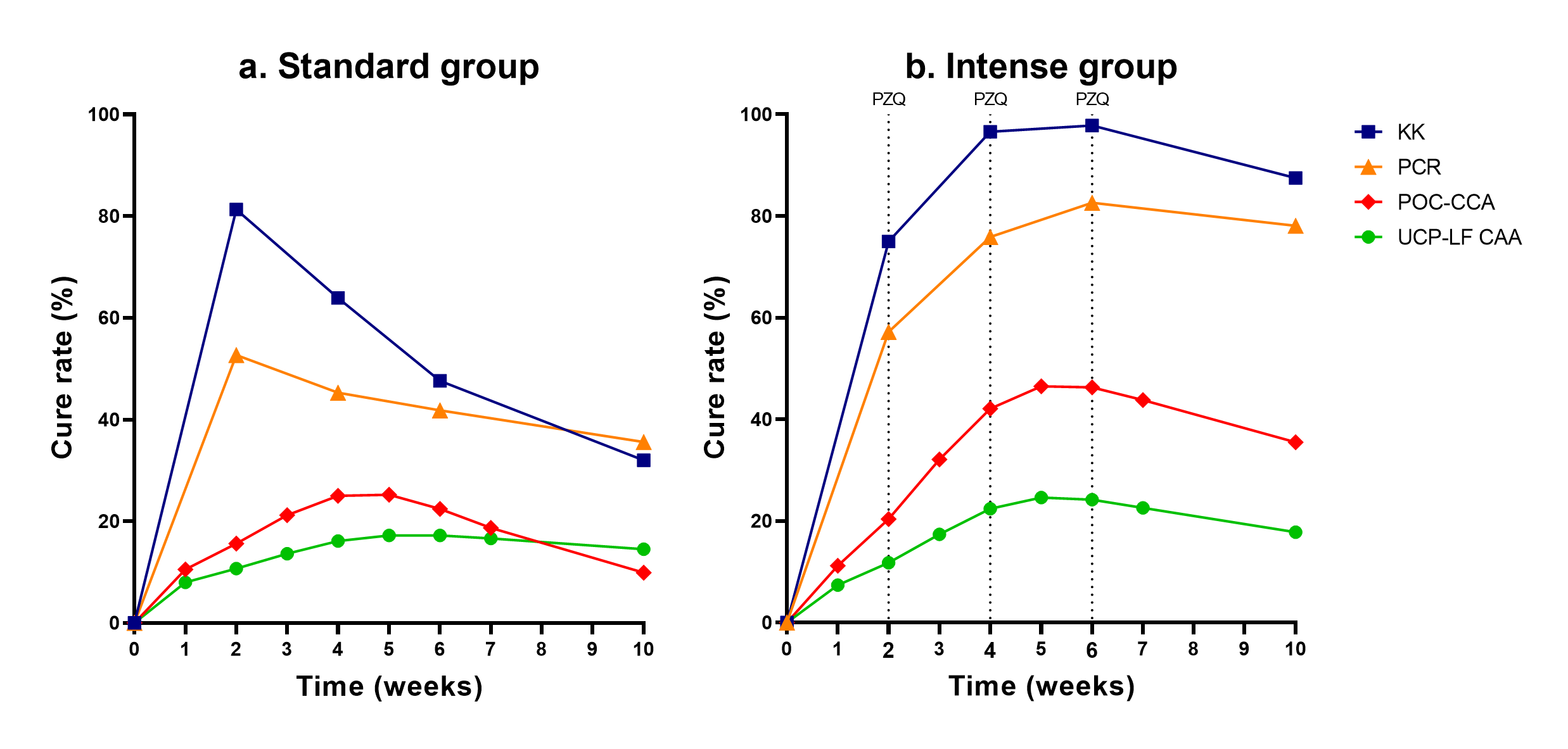

Supplement: S5 Fig — Cure rate (CR) over time determined by the different diagnostics in (a) the standard treatment group, who received a single dose of PZQ at week 0, and (b) the intense treatment group, who received four doses of PZQ at weeks 0, 2, 4, and 6. Data based on stool polymerase chain reaction (PCR), Kato-Katz (KK), urine up-converting particle circulating anodic antigen (UCP-LF CAA), and point-of-care circulating cathodic antigen (POC-CCA) (n = 125). (TIF) [file pntd.0011008.s005.tif]

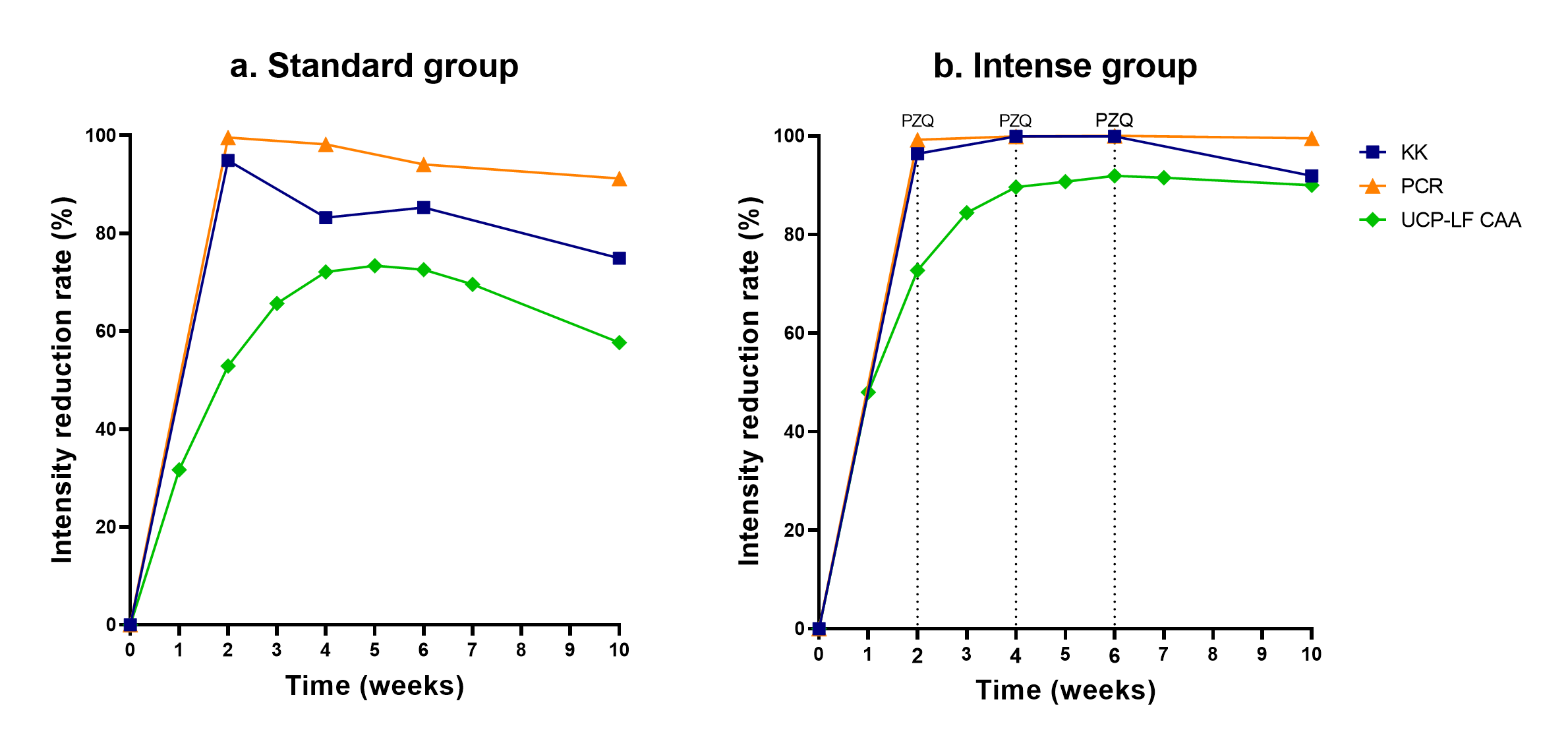

Supplement: S6 Fig — Intensity reduction rate (IRR) over time determined by the different diagnostics in (a) the standard group, who received a single dose of PZQ at week 0, and (b) the intense group, who received four doses of PZQ at weeks 0, 2, 4, and 6. Data based on stool polymerase chain reaction (PCR), Kato-Katz (KK), and urine up-converting particle circulating anodic antigen (UCP-LF CAA) (n = 125). (TIF) [file pntd.0011008.s006.tif]

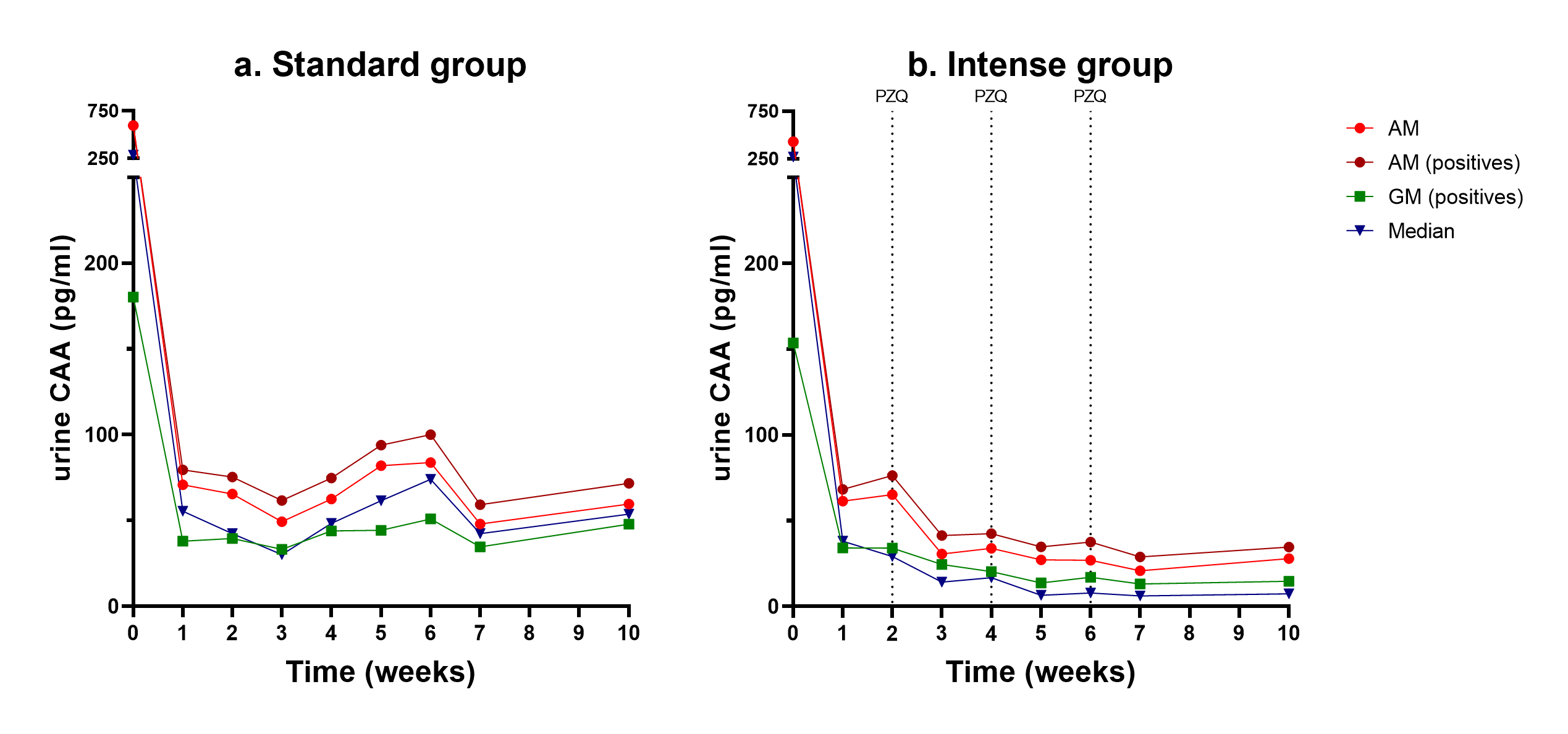

Supplement: S7 Fig — Circulating anodic antigen (CAA) levels over time in (a) the standard group, who received a single dose of PZQ at week 0, and (b) the intense group, who received four doses of PZQ at weeks 0, 2, 4, and 6. Data shown as arithmetic mean (AM), arithmetic mean of the positives, geometric mean (GM) of the positives, and median. (TIF) [file pntd.0011008.s007.tif]
