# Supplementary material for: Limited efficacy of repeated praziquantel treatment in Schistosoma mansoni infections as revealed by highly accurate diagnostics, PCR and UCP-LF CAA (RePST trial)
Source: PLoS Negl Trop Dis. 2022 Dec 22;16(12):e0011008. doi: 10.1371/journal.pntd.0011008 (PMC9822103; doi:10.1371/journal.pntd.0011008)
Supplement: S1 Table — Adapted from (24), for the current study based on data from 125 school-aged children. (DOCX) [file pntd.0011008.s008.docx]

**S1 Table. Baseline characteristics of the standard treatment group and the intense treatment group.** Adapted from (1), for the current study based on 125 school-aged children.

|  | **Standard treatment group**  (N=56) | **Intense treatment group**  (N=69) |
| --- | --- | --- |
| Age, years | 10.5 (9-12) | 10 (9-12) |
| Sex |  |  |
| Boys | 36 (64%) | 45 (65%) |
| Girls | 20 (36%) | 24 (35%) |
| Village |  |  |
| Ahouaty | 30 (53%) | 39 (57%) |
| N’Denou | 21 (38%) | 25 (36%) |
| Singrobo | 5 (9%) | 5 (7%) |
| Infection intensity |  |  |
| Kato-Katz |  |  |
| Light (1-99 EPG) | 14 (25%) | 26 (38%) |
| Moderate (110-399 EPG) | 25 (45%) | 32 (46%) |
| Heavy (≥400 EPG) | 17 (30%) | 11 (16%) |
| POC-CCA^a^ |  |  |
| 1+ | 11 (20%) | 17 (25%) |
| 2+ | 31 (55%) | 44 (64%) |
| 3+ | 14 (25%) | 8 (11%) |
| PCR |  |  |
| Low (35 ≤ Ct < 50) | 0 | 0 |
| Moderate (30 ≤ Ct < 35) | 1 (2%) | 3 (4%) |
| High (Ct < 30) | 55 (98%) | 66 (96%) |
| UCP-LF CAA |  |  |
| Low (0.6-9 pg/ml) | 5 (9%) | 7 (10%) |
| Moderate (10-99 pg/ml) | 12 (21%) | 12 (17%) |
| High (>100 pg/ml) | 39 (70%) | 50 (72%) |

Data are median (IQR) or n (%). Abbreviations: EPG, eggs per gram of stool; IQR, interquartile range; POC-CCA, point-of-care circulating cathodic antigen.

^a^ POC-CCA positive G-scores were classified into 1+ (G4-5), 2+ (G6-7) or 3+ (G8-10).

Reference

1. Hoekstra PT, Casacuberta-Partal M, van Lieshout L, Corstjens P, Tsonaka R, Assaré RK, et al. Efficacy of single versus four repeated doses of praziquantel against *Schistosoma mansoni* infection in school-aged children from Côte d'Ivoire based on Kato-Katz and POC-CCA: an open-label, randomised controlled trial (RePST). PLoS Negl Trop Dis. 2020;14:e0008189.
