# Supplementary material for: Limited efficacy of repeated praziquantel treatment in Schistosoma mansoni infections as revealed by highly accurate diagnostics, PCR and UCP-LF CAA (RePST trial)
Source: PLoS Negl Trop Dis. 2022 Dec 22;16(12):e0011008. doi: 10.1371/journal.pntd.0011008 (PMC9822103; doi:10.1371/journal.pntd.0011008)
Supplement: S2 Table — Data based on polymerase chain reaction (PCR), Kato-Katz (KK), up-converting particle circulating anodic antigen (UCP-LF CAA), and point-of-care circulating cathodic antigen (POC-CCA). (DOCX) [file pntd.0011008.s009.docx]

**S2 Table. Cure rate (CR) and intensity reduction rate (IRR) of a single (standard treatment group) and four (intense treatment group) repeated PZQ treatments in 125 school-aged children with a confirmed *S. mansoni* infection.** Data based on polymerase chain reaction (PCR), Kato-Katz (KK), up-converting particle circulating anodic antigen (UCP-LF CAA) and point-of-care circulating cathodic antigen (POC-CCA).

|  | **Standard treatment group**  **(N=56)** | **Intense treatment group**  **(N=69)** |
| --- | --- | --- |
| **PCR** |  |  |
| Cured children 4 weeks post-treatment | 25 | 52 |
| CR (95% CI)^a^ | 45.3% (32.3 – 58.8%) | 78.1% (66.4 – 86.6%) |
| Median AU^b^ |  |  |
| Before treatment | 32768 | 16384 |
| 4 weeks post-treatment | 2048 | 24 |
| Arithmetic mean AU^a^ |  |  |
| Before treatment (95% CI) | 5.6x10^5^ (2.3 x10^5^ – 1.2x10^6^) | 2.0x10^5^ (9.5x10^4^ – 4.2x10^5^) |
| 4 weeks post-treatment (95% CI) | 1.0x10^4^ (3.4x10^3^ – 2.8x10^4^) | 9.9x10^2^ (2.2x10^2^ – 4.3x10^3^) |
| IRR^a^ | 99.6% (98.6 – 99.9%) | 99.5% (97.2 – 99.9%) |
| **Kato-Katz** |  |  |
| Cured children 4 weeks post-treatment | 35 | 58 |
| CR (95% CI)^a^ | 63.9% (51.7 – 74.5%) | 87.5% (77.9 – 93.2%) |
| Median EPG^b^ |  |  |
| Before treatment | 204 | 136 |
| 4 weeks post-treatment | 0 | 0 |
| Arithmetic mean EPG^a^ |  |  |
| Before treatment (95% CI) | 288 (174 – 478) | 164 (102 – 264) |
| 4 weeks post-treatment (95% CI) | 48 (27 – 87) | 13 (5 – 33) |
| IRR (95% CI)^a^ | 83.2% (70.5 – 90.4%) | 91.9% (77.8 – 97.1%) |
| **UCP-LF CAA** |  |  |
| Cured children 4 weeks post-treatment | 9 | 13 |
| CR (95% CI)^a^ | 16.1% (10.5 – 24.0%) | 17.8% (11.5 – 26.3%) |
| Median urine CAA-level (pg/ml)^b^ |  |  |
| Before treatment | 286 | 270 |
| 4 weeks post-treatment | 61 | 14 |
| Arithmetic mean urine CAA-level (pg/ml)^a^ |  |  |
| Before treatment (95% CI) | 145.6 (93.3 – 217.8) | 153.4 (102.5 – 219.3) |
| 4 weeks post-treatment (95% CI) | 40.6 (25.2 – 62.2) | 15.2 (9.3 – 23.3) |
| IRR (95% CI)^a^ | 72.1% (62.4 – 79.4%) | 90.1% (86.9 – 92.5%) |
| **POC-CCA** |  |  |
| Cured children 4 weeks post-treatment | 16 | 24 |
| CR (95% CI)^a^ | 25.0% (17.2 – 34.9%) | 35.5% (25.5 – 46.9%) |
| Median G-score^b^ |  |  |
| Before treatment | 6.5 | 7.0 |
| 4 weeks post-treatment | 6.0 | 5.0 |
| Arithmetic mean G-score^a^ |  |  |
| Before treatment | 6.6 | 6.3 |
| 4 weeks post-treatment | 5.6 | 4.6 |
| IRR^c^ | 15.2% | 27.0% |

Abbreviations: AU, arbitrary unit (see Materials and Methods for definition); CAA, circulating anodic antigen; CR, cure rate; IRR, intensity reduction rate; PCR, polymerase chain reaction; POC-CCA, point-of-care circulating cathodic antigen; PZQ, praziquantel; UCP-LF, up-converting particle lateral flow.

1. Calculated from the model
2. Median of the positives
3. Calculated manually
