# Supplementary material for: Limited efficacy of repeated praziquantel treatment in Schistosoma mansoni infections as revealed by highly accurate diagnostics, PCR and UCP-LF CAA (RePST trial)
Source: PLoS Negl Trop Dis. 2022 Dec 22;16(12):e0011008. doi: 10.1371/journal.pntd.0011008 (PMC9822103; doi:10.1371/journal.pntd.0011008)
Supplement: Attachment — Submitted filename: 221121_RePST_diagnostics_Response to editor+reviewers.docx [file pntd.0011008.s010.docx]

**Reviewer 1**

"The manuscript "Limited efficacy of repeated praziquantel treatment in Schistosoma mansoni infections as revealed by highly accurate diagnostics, PCR and UCP-LF CAA (RePST trial)" aimed to evaluate PZQ efficacy using PCR and UCP-LF CAA in addition to the techniques already employed in this kind of study (KK and POC-CCA). However, the results did not show the relevance of the different methods used. Also, there is no relevant difference between the obtained data from KK and POC-CCA and PCR and UCP-LF CAA. Accordingly, I recommend rejecting the manuscript."

We thank the reviewer for their critical feedback. Microscopy is being criticized more and more because of its low(er) sensitivity, in particular in detecting low intensity infections which are often observed after treatment. Our study evaluated the efficacy of praziquantel treatment. Even though the majority of studies often still use microscopy (either KK for *S. mansoni* or urine filtration for *S. haematobium*), more accurate diagnostics are needed to determine the true efficacy of praziquantel. We have tried to emphasize this throughout the manuscript, starting in the abstract:

(Lines 46-48)

*‘To accurately evaluate PZQ efficacy, we employed more sensitive DNA and circulating antigen detection methods.’*

but also in the discussion:

(Lines 297-300)

*‘In this study, performing additional diagnostic analysis on samples previously collected at a multiple treatment trial comparing single versus 4 treatments with PZQ in school-aged children from Côte d’Ivoire, we confirmed our prior conclusion that highly accurate diagnostic methods play a crucial role in measuring PZQ efficacy (23).‘*

Our results showed and discussed the relevance of using worm-based diagnostics, such as POC-CCA (already previously: <https://doi.org/10.1371/journal.pntd.0008189>) and UCP-LF CAA (current manuscript). This study is the first to publish results on the efficacy of PZQ determined by egg-related diagnostic procedures as well as worm-related diagnostic procedures. Even though no eggs were detected after (repeated) treatment, it is not clear whether this is due to a cleared infection or if there are still worms present which could eventually start producing eggs again. Our study showed that most probably worms remain present, even after repeated treatment – based on detectable CAA levels in urine. From our study, we cannot be sure whether the antigen we are detecting originated from living worms or from dead and/or decaying worms. However, in travelers and migrants, who had a confirmed *Schistosoma* infection and were treated according to national guidelines, we observed a decrease in urine CAA-levels within days to weeks after treatment, indicating that – in the absence of reinfection – CAA-levels can become undetectable after successful treatment (Hoekstra et al., 2021 Travel Med Infect Dis and Hoekstra et al., 2022 Am J Trop Med Hyg).

This stresses the need to further investigate the relationship between excretion/detection of eggs and excretion/detecting of adult worm-related antigen, which has already been mentioned in lines 350-354 in the discussion:

*‘To further elucidate these mechanisms and to differentiate between surviving adult worms and establishing new infections (reinfection), research in endemic settings with various (seasonal) transmission patterns, in particular the absence of transmission, is needed. Alternatively, experimental infections in animals or controlled human infection models would be helpful in unravelling the exact metabolic aspects of CAA excretion and immune-mediated clearance patterns.’*

**Reviewer 2**

Yes, the proposed objectives are in accordance with the tested hypothesis. The study design is in accordance with the proposed objective. The sample used here originates from a previous publication and although it started from a very large group of schoolchildren and only a little more than 125 children were left for the analyses, even so this number is enough to carry out the statistical analyzes which support well the conclusions drawn. The authors present the necessary approvals to the local ethics committee and the institution responsible for carrying out the exams.

We thank the reviewer for their positive feedback. Indeed, the total number of children analyzed in the current manuscript (N=125) is somewhat lower than the previous publication (N=167). However, the number was still sufficient to perform statistical analysis: based on our sample size calculation, we needed at least 30 children per group to have enough power for our statistical analysis (see published study protocol, Hoekstra et al. 2018 BMC Infect Dis).

The results are well described and in accordance with the proposed objectives. The amount of tables and figures is enough to clearly show what is proposed. Although there is no gold standard for comparison, the authors determined for comparison the composite reference standard and considered the individual who was positive in the four tests truly positive.

Many thanks for the positives words.

Yes, the conclusions are supported by the data presented. The data obtained make a great contribution to the question of eliminating schistosomiasis as proposed in the WHO guidelines. It is clear that the determination of the cure rate requires a more complex analysis. More sensitive methods certainly showed if, in fact, these worms were truly eliminated or not. It remained a curiosity to know if the analyzes had been extended for another 2 weeks how this data would behave. I consider that these data are relevant for Public Health.

Indeed, in our manuscript we have tried to make clear that the cure rate very much depends on which diagnostic method is used to determine it. Of course it would be very interesting to see the outcomes for each diagnostic test 2 weeks later, or even 6 months or 1 year later. Unfortunately, this could not be done due to budget constraints, but it would have been very interesting to see whether infections levels would have turned to baseline levels – which is highly likely due to continuous exposure and ongoing transmission in the study region.

Although the authors refer to previous works, it would be important to make reference in the discussion (or even in the methodology) about the issue of rejection or even the difficulty of treating children with PZQ pills, speak also if in the intensive treatment group how they dealt with the adverse reactions that routinely occur. Another curiosity is to know if with so many evaluations (sample collections) there was no loss of individuals for not delivering the samples. A comment would be in order. They are minor modifications.

Many thanks for pointing this out. Indeed, this was a major part of our first publication where we reported data regarding the uptake and safety of PZQ. During the trial, we had a very dedicated study team, and several measures were put in place to make sure the children took the pills. For example, all children were given an appropriate meal before treatment to minimize side effects. Direct observed treatment was applied, and if a child refused, the team tried to mix the pills for example with some food or lemonade to increase the uptake. Also, in case a child vomited, another dose was given. All children stayed at the clinic for observation of side effects. No severe adverse events were observed. In the children who were treated repeatedly, the number of observed side effects decreased with every treatment.

Regarding the lost to follow-up, this was minimal thanks to our amazing and highly dedicated study team. Details regarding the very few that were lost to follow up can be found in the supplementary materials of our first study publication (<https://doi.org/10.1371/journal.pntd.0008189.s006>).

The current manuscript focuses on the evaluation of different diagnostic methods, and therefore we rather refer the reviewer to the first study publication (Hoekstra et al., 2020 PLOS NTD).

The data presented clearly warns us that we need to rethink the treatment of schistosomiasis, not only in terms of the dose to be applied, but also in the search for new drugs.

Dear Editor,

We really appreciate that you have taken the time to review our manuscript yourself, our sincere thanks. We would like to respond to your comments and questions, please find a detailed reply below.

In an effort to prevent any further delays to processing your submission, I have informally reviewed the article myself, particularly in order to see if I could understand this second reviewer's concerns. I think it would be helpful to see the comparisons you undertook in your supplementaries featuring more prominently in your manuscript in order to address these concerns. I certainly won't tell you how to design your study or author your manuscript, but I think it might be helpful if you considered a main text figure and/or table (both perhaps?) clearly demonstrating:

1. The differences in sensitivity of the four methods you employed and their relative agreement with your surrogate 'gold-standard'. You mention in your text that the UPC-LC CAA method has been shown in prior study to have 'high' specificity and sensitivity, but I think demonstrating this using your control material is important for reader's in interpreting the PZQ CR results. I understand that this is in your supplementaries, but I think it might be better placed in the main text.

In your feedback, you mention several times to include (some of the) supplementary materials in the main text. We are not sure whether we understand exactly what is meant by this, in particular with the sentence ‘demonstrating this (ie sens/spec) using your control material is important for reader's in interpreting the PZQ CR results’. We mention that the UCP-LF CAA test has a high sensitivity and specificity, which is based on numerous previous studies which have already been included in the list of references of our manuscript. Indeed, in the final paragraph of the results we briefly mention the accuracy of the diagnostic methods. As this was not the main objective of the study, we initially thought it would be better to include the table as supplementary material, especially since it does not only describe the accuracy of each test pre-treatment but also after treatment. However, we acknowledge that this might be relevant information and therefore, it is now included as ‘Table 2’ in the manuscript rather than ‘Supplementary Table 2’.

2. Similarly, you demonstrate in the supplementaries, as notable difference in the estimated CR based on the four methods you have used, but again, I think having this as a main figure and highlighting which of these differences are statistically significant would be helpful to the reader.

Indeed, in Supplementary Table 3 we provide an overview of the CR based on all four diagnostic methods. However, as the focus of the current manuscript is on PCR and UCP-LF CAA and outcomes based on KK and POC-CC already have been published previously, we decided to only include the CR and IRR based on PCR and UCP-LF CAA in the main text of the manuscript. However, we have included Supplementary Table 3, in case the reader would like to compare the CR and IRR from all diagnostic methods applied in the study. A reference to Supplementary Table 3 has been included in the text.

2. Finally, do you have data for the relative diagnostic specificity of each method? Particularly specificity for patent/live infections.

The assumed specificity of each diagnostic method is based on previous studies/publications. For microscopy, in general a specificity of 100% is assumed (‘an egg is an egg’). Regarding POC-CCA, specificity is assumed to be high, but some non-specific signals have been observed and reported by others as well. This has also been discussed in our previous publication which focused on KK and POC-CCA results from the same trial. This is the main reason why the POC-CCA was not part of the composite reference standard.

The current manuscript focuses on PCR and UCP-LF CAA: PCR has a confirmed specificity of 100%, based on technical evaluations, as well as from the HEMQAS participation (lines 152-155). Similarly, the UCP-LF CAA test is also assumed to be 100% specific, as the antigen, CAA, is found to be 100% specific due to its unique chemical structure. It is found only in schistosomes and has not been observed elsewhere. As both CCA and CAA are regurgitated by the worms, both antigens are a marker for active infection. References for this are already included in the manuscript.

With the antigen tests, is there any way to know for how long hosts will continue to shed worm antigens after the worms themselves have been killed through PZQ treatment?

In previous studies, using CAA to measure the effect of treatment, it has been shown that the antigen levels significantly decrease within days to weeks after treatment (eg van Lieshout et al., 1997 Trop med Int Health; van Grootveld et al., 2018 Eur J Clin Microbiol Infect Dis; Sousa et al., 2019 Front Immunol; Langenberg et al., 2020 Nat Med; Hoekstra et al., 2021 Travel Med Infect Dis; Tamarozzi et al., 2021 Parasit Vectors). However, as observed in the current study, antigen levels do not seem to further decrease after repeated treatment. This could mean that (some) worms, including possibly young worms, are not affected by PZQ and continue to excrete CAA. Also, in our study setting, reinfection cannot be ruled out either, so CAA could also come from recently acquired young worms. All these different possibilities have already been included in the discussion, see lines 333-359. A different study set up would be required to shed more light on the different potential mechanisms explaining our findings.

Similarly, is there any data that following the apparent cessation of egg production after PZQ (inferred by KK for example) that hosts still showing infection based on UPC-LC CAA or POC-CCA eventually resume egg-shedding (as evidence of the survival of any remaining worms and their return to reproductive fecundity). I presume such data would have to come from a hamster or similar model animal.

This would indeed be very interesting to investigate. In our study, in particular in the control group, we observed a decline in the number of eggs detected after treatment. However, over the course of a few weeks the number of eggs detected started to increase again. The most likely explanation for this would be that young schistosomula known to be refractory to PZQ, continue to develop and start to produce eggs. In case of newly contracted reinfections, it generally takes longer for eggs to be excreted. Damaged but recovered adult worms may also contribute to egg excretion again. At the final time point, 10 weeks after the first treatment, it could be a combination of new infections and ’recovered’ infections. Indeed, an animal model would be a good approach to investigate this further. On the other hand, it is generally acknowledged that small rodent animals do not fully mimic the human situation, e.g. in body size, while studies with non-human primates are almost impossible to perform.

I think this is particularly worth considering in the intense treatment group in whom you measured a substantial reduction in infection based on KK, but much less so with the other methods after week 2 or so. Could it be that either treatment has sterilised the surviving adults (as has been seen in, for example, canine heartworm treatment)? Again, is their data that can refute concerns that what you are seeing is prolonged antigen shedding from dead and decaying worms and not live adults?

We are not sure whether we understand your point regarding the substantial reduction in infection based on KK in the intense treatment group. In Figure 2 we show that based on all quantitative diagnostic methods (KK, PCR on stool, and UCP-LF CAA on urine), infection levels decrease significantly after a single treatment. Infection levels further decrease when repeating treatment, as demonstrated by KK and PCR on stool, and to a smaller extent also by UCP-LF CAA in urine. Some antigen we are detecting would certainly be coming from dead and decaying worms, but the prolonged antigen levels can only be explained by the continuing or newly presence of young and adult schistosomes. In travelers and migrants with a confirmed *Schistosoma* infection and who were treated according to national guidelines, we observed a decrease in urine CAA-levels within days to weeks after treatment, indicating that – in the absence of reinfection – CAA-levels can become undetectable after successful treatment (Hoekstra et al., 2021 Travel Med Infect Dis and Hoekstra et al., 2022 Am J Trop Med Hyg).
